# Complete Genome Sequence of *Salmonella enterica* subsp. *enterica* Serovar Thompson Strain RM6836

**DOI:** 10.1128/genomeA.00900-13

**Published:** 2013-11-14

**Authors:** Craig T. Parker, Lisa Gorski, Steven Huynh

**Affiliations:** Produce Safety and Microbiology Research Unit, Western Regional Research Center, Agricultural Research Service, U.S. Department of Agriculture, Albany, California, USA

## Abstract

*Salmonella enterica* subsp. *enterica* serovar Thompson strain RM6836 was isolated from lettuce in 2002. We report here the complete sequence and annotation of the genome of *S.* Thompson RM6836. This is the first reported complete genome sequence for *S.* Thompson and it will enhance our understanding of this serovar and provide another point for comparative studies between *Salmonella enterica* strains.

## GENOME ANNOUNCEMENT

*Salmonella enterica* subsp. *enterica* is a major cause of food-borne illnesses associated with a wide variety of foods, including meat, eggs, fruits, vegetables, nuts, and spices. Based on the serologic identification of O (lipopolysaccharide) and H (flagellar) antigens, *S. enterica* subsp. *enterica* has been classified into a variety of groups and specific serovars (1). Among *S. enterica* subsp. *enterica* strains, the most common O-antigen serogroups are A, B, C1, C2, D, and E, and these serogroups cause approximately 99% of *Salmonella* infections in humans (1). *S. enterica* subsp. *enterica* serovar Thompson is in serogroup C1 and has been the cause of food-borne outbreaks associated with cilantro, arugula, chicken, beef, bread, and smoked salmon (2, 4–8). *S.* Thompson strain RM6836 was isolated from lettuce in 2002 and serotyped by the FDA Center for Veterinary Medicine as part of the USDA, Agricultural Marketing Service, Microbiological Data Program.

Genome sequencing was performed using shotgun and paired-end (8 to 12 kb) libraries and was generated on a Roche 454 FLX+ sequencing system with Titanium chemistry. The Roche Newbler assembler (version 2.3) was used to assemble 187,876 shotgun and 103,498 paired-end reads into 64 contigs and a single scaffold. Genome closing utilized a combination of steps. The contigs were aligned to the other genomes of *S. enterica* subsp. *enterica*, including serovar Typhimurium LT2 and serovar Enteritidis strain P125109, using the software Mauve (3) to find unexpected gaps. Scaffold gaps were filled by a combination of referenced assemblies of 1,907,370 Illumina MiSeq reads to the Newbler contigs using Geneious version 6.1.6 and the identification of repeated contigs using the Perlscript contig_extender2. Certain gaps were validated using PCR amplification and Sanger sequencing. All base calls were validated using the Illumina MiSeq reads, which provided an additional 100× coverage.

The *S.* Thompson RM6836 genome size is 4,707,648 bp, with a G+C content of 52.2%. The genome sequence was annotated using the NCBI Prokaryotic Genomes Automatic Annotation Pipeline (PGAAP) (http://www.ncbi.nlm.nih.gov/genomes/static/Pipeline.html) and was deposited with GenBank. The RM6836 genome is predicted to carry 4,621 genes, 7 ribosomal RNA operons, and 79 tRNAs. Bacteriophages were identified using PHAST (9), including one identified as Gifsy 1 and four remnant prophages. The *S.* Thompson RM6836 genome is highly syntenic to other *S. enterica* subsp. *enterica* serovars, with variable positions of prophage and bacteriophage remnants in the different serovars. RM6836 does not possess a virulence plasmid, which is common to many other *S. enterica* subsp. *enterica* strains. To our knowledge, this is the first complete *S.* Thompson genome sequence to be released into the public domain.

### Nucleotide sequence accession number.

The *S.* Thompson RM6836 genome sequence has been deposited in GenBank under the accession no. CP006717.
